# Medical complications of obesity: heightened importance in a COVID era

**DOI:** 10.1186/s12245-022-00431-7

**Published:** 2022-06-23

**Authors:** Heather Prendergast, Carissa Tyo, Christopher Colbert, Morgan Kelley, Ruth Pobee

**Affiliations:** Department of Emergency Medicine, University of Illinois at Chicago, Chicago, IL USA

**Keywords:** Obesity, Type 2 diabetes, Hypertension, Dementia, Non-alcoholic fatty liver disease, Polycystic ovarian syndrome, Dyslipidemia, Cancer

## Abstract

**Background:**

Obesity is a major public health problem associated with significant medical complications.

**Main body:**

This review examines 8 primary diseases: type 2 diabetes, hypertension, dementia, non-alcoholic fatty liver disease, polycystic ovarian syndrome, dyslipidemia, cancer, and their manifestations in obese patients. A total of 39 articles were used for this review. The authors conducted limited review, searching PubMed and Google Scholar databases using a combination of key words “COVID-19” or “SARS-COV2”, “type 2 diabetes”, “hypertension”, “dementia”, “non-alcoholic fatty liver disease”, “polycystic ovarian syndrome”, “dyslipidemia”, “cancer”, and “obesity”. No specific date limitation was used. Obesity exacerbates many medical conditions and has recently been identified as an independent risk factor for COVID-19 severity. This sets obesity at the pinnacle of all disease complications. The long-term impact of obesity ranges from financial burden on the health system, lower life expectancy, and reduced survival rates.

**Conclusion:**

Obesity is an important modifiable risk factor. There is the need for healthcare providers to understand the medical complications associated with obesity to optimize patient care.

## Background

Obesity is a worldwide epidemic affecting people from different countries and of all ages. The World Health Organization (WHO) reported that obesity prevalence nearly tripled between 1975 and 2016 [1]. In 2016, more than 1.9 billion adults aged 18 years and older were overweight. Of these, over 650 million adults were obese [1]. If current trends continue, it is estimated that 2.7 billion adults will be overweight, over 1 billion obese, and 177 million severely obese by 2025 [2]. Obesity has caused a substantial financial burden on the healthcare system [3]. As a result of the increasing prevalence, obesity is associated with the development of medical complications such as type 2 diabetes, hypertension, dementia, non-alcoholic fatty liver disease, polycystic ovarian syndrome, dyslipidemia, and cancer.

Obesity has been associated with increase mortality, with nearly 1 in 5 deaths attributable to obesity, a number that is 3 times previous estimates [4]. The obesity epidemic is threatening over a century of gains in life expectancy and is now a significant contributor to relatively low life expectancy among high-income countries. In fact, studies show that the more recent the birth year, the greater effect obesity has on mortality rates [4]. The estimated annual number of deaths attributed to obesity in the USA is in excess of 280,000 [5]. People aged 35–59 years with morbid obesity (body mass index (BMI) 40–50) were 5 times more likely to die from ischemic heart disease and 6.5 times at risk of dying from stroke and 22.5 times at risk of dying from diabetes compared with people with lower BMI [5]. The impact of morbid obesity on health is so severe that median survival of affected people reduced by about 8 to 10 years [5].

Furthermore, the recent surge of the COVID-19 pandemic identified obesity as an independent risk factor to COVID-19 severity and complications making obesity a predominant problem associated with numerous medical complications [6–10].

Researchers have identified eight primary diseases related to metabolic dysfunction and obesity is a marker for all of them. In fact, these eight primary obesity-related diseases account for a staggering 75% of healthcare costs in the USA [11]. Obesity has been identified as one the most important modifiable risk factor aside from smoking [12]. Obesity contributes to multisystem pathology; therefore, awareness is necessary in order to formulate appropriate evaluation and management plans when treating the obese patient. As our understanding of the pathophysiology associated with metabolic syndrome has improved, it is important to note that while obesity is a marker for metabolic syndrome that it is not the cause [13].

This review will focus on the eight leading primary diseases associated with obesity and metabolic dysfunction. We will begin with role of obesity and the COVID-19 pandemic.

The authors conducted limited review, searching PubMed and Google Scholar databases using a combination of key words “COVID-19” or “SARS-COV2”, “type 2 diabetes”, hypertension, dementia, non-alcoholic fatty liver disease, polycystic ovarian syndrome, dyslipidemia, cancer and “obesity”. We selected articles published exclusively in English. We evaluated systematic reviews, meta-analyses, retrospective and prospective studies, and other narrative.

reviews. A total of 39 articles were reviewed without any specific date limitation for our search.

## Main text

### COVID-19 infection and obesity

Since the start of the COVID-19 (SARS-CoV-2) pandemic, public health officials, epidemiologist, and medical officers have been working tirelessly towards a solution to control and curb this pandemic. Risk factors that were quickly identified among people infected with SARS-CoV-2 include hypertension, diabetes and other cardiovascular diseases [14]. Obesity was subsequently identified as an independent risk factor to COVID-19 and was placed on the Centers for Disease Control (CDC) high risk criteria [15]. Since then, several retrospective studies from different countries and systematic reviews have been conducted to examine the prevalence and severity of SARS-CoV-2 among obese patients.

A high prevalence of overweight (48%) and obesity (21–46%) exist among COVID-19 patients experiencing adverse complications in many countries [6, 16–21]. A New York study found nearly half (46%) of its population of critically ill COVID patients were obese (BMI ≥ 30 kg/m^2^) [16] while a study from Detroit, found 38% of obesity among African Americans [19] and in France, 67% of its critically ill COVID-19 patients had excess body weight [21]. Similarly, a systematic review involving eight retrospective studies found a higher risk of severe illness among obese patients older than 60 years [22]. On the contrary, a study in Greece found lower rates of obesity in the elderly population, with 34% of obesity in ICU patients younger than 55 years [23].

Obesity is also associated with progression to severe COVID-19 in adults hospitalized with SARS-CoV-2 infection [6, 24–26], with more severe COVID-19 infection among obese than non-obese patients. Similarly, in a large prospective cohort of 502,543 middle aged adults in the UK, both BMI and waist circumference were associated with testing positive for COVID-19 in a dose–response fashion [27]. Likewise, a case series study in China found obesity in men significantly increased the risk of developing severe COVID-19 than in women [28]. The association between obesity and COVID infection has been linked to the negative role of obesity in respiratory function. In obese patients, respiratory physiology is altered, resulting in decreased functional residual capacity and expiratory reserve volume leading to subsequent ventilation-perfusion abnormalities and hypoxemia [29]. However, these mechanisms are still not fully elucidated. Thus, prevention of obesity remains key to the progression to more severe forms of COVID.

### Type 2 diabetes and obesity

Diabetes mellitus, a chronic degenerative metabolic disease, has reached epidemic proportions within the last three decades and is directly linked with the obesity epidemic [30, 31]. Ninety percent of patients with type 2 diabetes have a BMI > 23. Type 2 diabetes affects 28 million people in the USA. Even more worrisome are the more than 80 million individuals who are classified as prediabetic [13]. The long-term complications of diabetes are responsible for significant morbidity and mortality and are the direct result of the associated microvascular disease. Microvascular disease has been shown to accelerate the development of heart failure, contribute to atherogenesis, cause renal dysfunction, retinopathy, and neuropathy [13]. Most of the economic burden associated with diabetes is attributed to the disease complications. Diabetes and the resultant sequelae account for more adult cases of end-stage renal disease, limb amputations, and vision loss than any other disease [13].

Elevation of blood glucose occurs in most individuals with obesity and is categorized as either prediabetes or diabetes. Prediabetes is defined as a fasting glucose in the range of 100–125 mg/dl, a 2-h postprandial level of 140–199 mg/dl, or hemoglobin A1C 5.7–6.4%. Categorical diabetes is defined as a fasting glucose > 126 mg/dl, or a postprandial level > 200 mg/dl [31]. While the primary cause of hyperglycemia in patients with obesity is insulin resistance, hyperglycemia is not the first indication of metabolic syndrome but develops as later sequelae [13].

Efforts to reduce the burden of microvascular and cardiovascular disease have started to show promise in that the annual incidence of diabetes may be decreasing for the first time in 3 decades in the USA [31].

It well documented that obesity management can delay progression from prediabetes to type 2 diabetes as well as demonstrated benefit in treatment of type 2 diabetes. In addition to first line therapy of weight loss and increased physical activity, Metformin therapy has be shown to slow conversion of prediabetes to diabetes [31]. Bariatric surgery may be considered for adults with BMI > 35 kg/m^2^ and type 2 diabetes who have not had a favorable management to lifestyle and pharmacological therapy.

### Hypertension and obesity

Hypertension in the context of obesity carries a greater potential for cardiovascular disease than any other risk factor. Seventy percentage of obese women with hypertension have evidence of left ventricular hypertrophy, a marker of subclinical heart disease [5]. The Asia–Pacific Cohort Collaboration Study demonstrated that for each unit change in BMI there was an associated 9% increase in ischemic heart disease-related events. The INTERHEART study found that abdominal obesity is significantly related to acute myocardial infarction [5].

Obese patients are 5 times more likely to suffer from hypertension and 85% of hypertension occurs in patients with a BMI > 25 [5]. Several mechanisms have been proposed to explain the relationship between hypertension and obesity. Contributing factors include (1) enhanced renal absorption of sodium secondary, (2) intravascular volume, (3) activations of the renin–angiotensin–aldosterone system and sympathetic nervous system, (4) release of angiotensinogen from adipose tissue, and (5) insulin resistance [13].

Within the last decade there have been significant increases in cases of adolescent hypertension. The upward trend in adolescent hypertension has been linked to the dramatic increases in adolescent obesity. The strongest correlation between body mass index and blood pressure in seen in adolescents classified as overweight or obese [32]. Most concerning about this increasing incidence of primary hypertension in the adolescent patient is the impact on cardiovascular risk and mortality (estimated at 12.8% worldwide) in adulthood. Risk factors (non-modifiable and modifiable) associated with a higher likelihood of developing hypertension included family history of hypertension, low birth weight, non-white ethnicity, low levels of physical activity and poor sleep regimen [32].

Intervention for adiposity-related hypertension in adolescence may partially mitigate some of the cardiovascular risk later in adulthood. Resolution of adolescence hypertension decreases the likelihood of developing subclinical heart disease. Studies have shown that reversal of adolescence obesity prior to adulthood is associated with a lower likelihood of developing hypertension in adulthood. Resolution of elevated BP prior to adulthood is associated with further risk reduction. Some studies have suggested that adolescence is the optimal period for preventing permanent cardiovascular damage.

Treatment of hypertension in the obese adults has shown definite benefit for long-term morbidity and mortality related to cardiovascular disease. Overall, research suggests that caloric restriction is a fundamental therapy/adjunct for hypertension management in the obese adult [13]. Successful examples of hypertension control have been demonstrated in individuals undergoing bariatric surgery, and following the Dietary Approaches to Stop Hypertension (DASH) diet. Regarding selection of anti-hypertensive medications, medications that do not induce insulin resistance or other metabolic syndrome risk factors are preferred. Agents fitting into this category (in order of preference) include angiotensin-converting enzyme (ACE) inhibitors or ARB (angiotensin II receptor blocker), non-selective beta/alpha-1 receptor blockers, and calcium channel blockers. In contrast, agents that have been associated with enhancing insulin resistance include beta-1 blockers and thiazide diuretics and should be avoided in the obese individuals [13].

### Dementia and obesity

Age–related dementia reflects a combination of cerebrovascular and pathogenic factors and results in progressive cognitive decline in the elderly [33]. Currently this incurable disease affects an estimated 35.6 million people worldwide and is trending to become a major health epidemic. There is increasing evidence that vascular risk factors occurring during midlife correlate with late-life dementia. Meta-analysis shows a moderate association between obesity and the risks of dementia and Alzheimer’s disease (AD) [34]. Alzheimer’s disease is the most common cause of progressive dementia in the elderly population with a disease prevalence of one in nine elderly age 65 or greater [34]. Studies show that obesity may precede dementia and that lifestyle factors play a critical role in the onset of AD. Overweight or obesity in middle age has been identified as an important and independent risk factor for later development of AD [34].

Investigation into the role of adipose tissue in the health of the brain has identified an association adipokines and cognitive decline. Adipokines (which means adipose cell in movement) affects processes in both the peripheral and central nervous system. Leptin, which is actively transported across the blood–brain barrier, has a notable effect on hippocampal development and function, predominately learning and memory processes. Some researchers have proposed that Leptin, an adipokine, may be a better predictor of dementia/mild cognitive impairment than standard anthropometric measures. One study found a greater association between higher serum leptin levels and a lower frequency of dementia in women with normal BMI’s as compared to obese women [34]. In addition, Hazzouri and colleagues found that obesity interferes with the neuroprotective effect of leptin on the brain leading to leptin resistance [34]. While some additional studies have confirmed that variation in leptin levels can be a risk factor associated with the development of AD, other studies failed to show a consistent role of leptin in AD pathology; therefore, the role of leptin remains controversial.

Despite the debate regarding the roles of certain adipokines, the association between midlife obesity and age-related dementia appears solid and complex. The literature identifies midlife obesity as a contributor and increased risk factor for developing dementia while the late–life weight loss signals impending dementia [33]. Researchers are now equipped to investigate new diagnostic tools and novel drug targets against age-related dementias.

### Dyslipidemia and obesity

As with many conditions induced or affected by obesity, lipid metabolism undergoes an unfavorable shift in patients with metabolic syndrome, and most particularly visceral obesity. Changes in the overall pattern of the lipid profile include elevated triglycerides (TG) and free fatty acids (FFA) with decreases in high density lipoprotein-C (HDL-C) and impaired HDL function along with increased small dense low density lipoprotein (LDL) [35]. With elevated fasting and post prandial TG the entire milieu of subsequent lipid metabolism shifts toward the creation of these smaller denser LDL particles. Paired with insulin resistance, these changes in the lipid profile generate a highly atherogenic environment.

In the post prandial state, rises in insulin levels generate increased inhibition of hormone sensitive lipase, a key factor in the generation of lipolysis of intracellular lipids. Increased FFA uptake in the adipocytes and myocytes occurs though a portion remains in the plasma where the FFA are bound by albumin and transported to the liver. Increased accumulation of TG in the liver leads to formation of VLDL and directly competes with the subsequent metabolism of chylomicrons by lipoprotein lipase (LPL) thereby further increasing remnant TG transportation to the liver. Persistent hypertriglyceridemia induces a shift in the exchange of cholesterol esters and TG with the resultant skewed profile of decreased HDL-C and decreased TG content in LDL which under the action of hepatic lipase produces increased amounts of low-density LDL. This low-density LDL is slowly metabolized resulting in increased atherogenicity.

Management of dyslipidemia in obesity revolves around a combination of lifestyle and dietary modifications. Total fat and calorie ingestion relates directly to postprandial lipemia and is the most direct target for management of the skew in lipid profile. Weight loss alone results in decreased LDL with increased LPL activity. The type of ingested fat also affects the postprandial lipemia favoring diets low in carbohydrate and high in monounsaturated fatty acids. Exercise has been theorized to favorably affect the lipid profile with increased LPL activity and TG metabolism [3]. Decreased TG levels have clearly been demonstrated though the effect on HDL-C levels remains controversial.

Beyond diet and exercise, pharmacological intervention may be required to address persistent dyslipidemia. Statins remain first line of therapy for patients having a favorable effect on LDL and non-HDL-C profiles. However, the principal driver of the dyslipidemias appears to arise from the shifts in TG metabolism and statins have little effect on the TG profile. Combination therapy with a fibrate or niacin may be considered in select populations with additional comorbidities like diabetes or known cardiovascular disease.

### Non-alcoholic fatty liver disease and obesity

Having described the overall shift in lipid metabolism, triglyceride (TG) excess arises as a factor in the development of hepatic steatosis as well [36]. Prevalence data suggest a direct corollary between Body Mass Index (BMI) and the degree of both hepatic steatosis and steatohepatitis. In the non-obese with a BMI < 30 kg/m^2^ the rates are 15% and 3% respectively. The rates to 65% and 20% respectively in patients with a BMI between 30 and < 40 kg/m^2^ and rise again to 85% and 40% respectively in patients with a BMI ≥ 40 kg/m^2^.

Free fatty acid (FFA) metabolism is a key driver of the accumulation of intrahepatic TG (IHTG). Circulating FFA arrive in the liver through both the hepatic artery and portal vein. Lipolysis of visceral adipose tissue contributes to the burden of FFA to a much smaller degree than subcutaneous fat metabolism [36]. Therefore, the amount of FFA presented to the liver rises with BMI. For those patients demonstrating signs of insulin resistance, the degree to which the liver produces its own FFA becomes a significant contributing factor. Insulin-resistant skeletal muscle shifts metabolism of carbohydrate away from the creation of intramuscular glycogen stores and back toward the liver resulting in increased FFA production and therefore IHTG accumulation.

Beyond the increased circulating FFA burden, patients with NAFLD demonstrate higher gene expression of hepatic lipase and hepatic lipoprotein lipase thereby augmenting TG metabolism within the liver directly. Other genetic determinants likely also contribute with higher expression of proteins involved in the uptake of FFA from plasma to tissue in obese patients demonstrating higher IHTG than obese patients with more normal IHTG.

The combination of high circulating FFA, increased IHTG metabolism and genetic determinants are believed to drive the presence of hepatic steatosis. The correlation of this increased IHTG burden and insulin resistance is less clear however. Whether insulin resistance is a by-product of the increased accumulation of intrahepatic FFA or a marker of another process driving both pathologies has proven challenging to discern. Regardless, when present together, the trend toward NAFLD rises.

NAFLD demonstrates a wide spectrum of disease from hepatic steatosis to non-alcoholic steatohepatitis (NASH) all the way through to frank cirrhosis. Identifying patients along this spectrum is crucial to mitigating risk of liver failure but remains challenging in the absence of liver biopsy. Abnormal transaminases may be an indicator of NASH, however, waiting until the markers shift may lead to underdiagnosis of NAFLD. A combination elevated transaminases and imaging showing increased fatty infiltration may be useful in the diagnosis of NASH, but not helpful in the staging of such disease. Once liver inflammation has progressed to cirrhosis, the degree of hepatic steatosis declines, and the liver becomes irrevocably damaged.

Management of NAFLD is tied directly to weight loss. A decline of as little as 5–10%, results in decreased hepatic steatosis and decreased steatohepatitis with improvement in biomarkers such as transaminases. These patients should be advised to avoid other hepatotoxins like alcohol and excess acetaminophen ingestion [37]. Co-management of other contributing disease like Diabetes may help to control the degree of hepatic steatosis. Once it has progressed to cirrhosis, these patients will require management similar to other causes of liver failure.

### Polycystic ovarian syndrome and obesity

The relationship between obesity and reproductive abnormalities remains complex. The presence of increased adipose tissue has an effect on circulating hormone levels. The distribution of this adipose tissue is directly affected by the hormonal milieu of the patient with higher testosterone levels driving a more android distribution of fat deposition to the upper body. It is no wonder then that the interplay of obesity and hormone effect can have a direct impact on fertility.

Polycystic ovarian syndrome is the most common endocrine disorder of reproductive age women occurring in ~ 7% of that group [38]. The syndrome is characterized by the presence of increased androgen production with abnormal gonadotropin hormone levels resulting in chronic anovulation and menstrual irregularity, infertility, and hirsutism. In addition to the reproductive effects, polycystic ovary syndrome (PCOS) patients tend to demonstrate other metabolic disorders with a tendency toward insulin resistance and type II diabetes [38]. Obesity is closely correlated with these findings and 40–80% of PCOS patients are reported to be overweight or obese. While the studies do not support a causal relationship between obesity and PCOS, they do demonstrate a corollary and support screening of obese patients for PCOS. Once present, PCOS may have an effect on the distribution of adipose tissue diverting deposition to areas above the waist with the attendant risk associated with visceral fat deposition to follow.

As with many diseases, weight loss has been shown to aid in the management of PCOS with additional attention directed to the treatment of insulin resistance and chronic anovulation.

### Cancer and obesity

The relationship between weight and cancer risk shows a clear horseshoe correlation. Patients at the extremes of the weight spectrum have an increased risk of malignancy. On the low end, the presence of a malignancy and/or risk factors for malignancy likely drive the presence of the low Body Mass Index (BMI). On the high end, obesity itself is believed to drive the risk of malignancy for a significant number of cancers with contributions to the development of breast, uterine, colon, kidney, gallbladder, pancreas, and esophageal cancer [39]. Elevated BMI further has a mortality effect that reaches beyond contributing to risk of development. For several cancers, the mechanism for the increased risk has been investigated and causes surmised.

In women, the risk of malignancy trends with the excess production of estrogen that arises from adipose tissue. Post-menopausal weight gain correlates directly with malignancy risk for both breast and endometrial cancer. Elevated circulating estrogens have been shown to increase risk for post-menopausal breast cancer with further support of the cause and effect relationship arising from use of estrogen receptor modulators to show decrease in the incidence. A similar mechanism is purported to contribute to the risk of endometrial cancer in conjunction with chronic anovulation.

Both men and women demonstrate increased risk of gastrointestinal tract pathology. Esophageal cancer rates trend higher in the obese believed to arise from effects of chronic reflux esophagitis and local inflammation. Colon cancer as well trends higher with one mechanism believed to contribute to this increased risk through chronic insulin resistance and effects of insulin-like growth factor. Pancreatic malignancy is also believed to arise from shifts in insulin production. Gallbladder malignancy is purported to increase related to chronic over-secretion and higher incidence of gallstones with chronic local inflammation. And finally, liver cancer appears to correlate with the increased incidence of NASH and cirrhosis noted in the obese.

Beyond the direct risk that BMI confers on patients for the development of cancer, obesity also contributes to challenges in the diagnosis and management of these patients [40]. In some circumstances, cancers may have improved detection by virtue of these patients having increased encounters with the medical establishment with more blood testing performed to manage ongoing disease and imaging studies performed to investigate other medical complaints. In other circumstances, patient size contributes to decreased success of screening and detection. Increased intra-abdominal fat may facilitate computed tomography imaging of the abdomen showing increased inflammatory response at the site of pathology [40]. However, this same increased girth results in greater attenuation in nuclear imaging studies thereby decreasing effectiveness of positron emission tomography or other nuclear studies. Physical exam itself may be impeded by excess body mass. Either through direct prevention of tactile discrimination or through physical impediments like sufficient table size, table weight limits, or tool size, the excess body mass may prevent the patient and physician from detecting a problem at its nascent phase.

Obesity poses a whole host of other challenges in post-diagnosis management as well. Dosing strategies for chemotherapy and radiation therapy are challenging in the morbidly obese often resulting in under-dosing with insufficient treatment for chemotherapeutics and risk of overdosing to achieve penetration for radiation therapy thereby increasing radiation exposure and risk for de novo malignancy. Operative challenges in the obese pose another contingent of complications in the management of malignancy in the obese population and are beyond the scope of this chapter.

Management for obese patients as it pertains to cancer follows two lines of thought. For those at risk for malignancy, weight loss has been shown to decrease risk for several malignancies. Recent bariatric surgery studies have shown a decreased risk of obesity-related cancers. Observational studies also suggest that postmenopausal weight loss decreases risk of breast cancer. After diagnosis, the data for impact of weight reduction is not as strong. Evidence suggests that weight gain after diagnosis is detrimental. However, weight loss after diagnosis is muddled by effects of treatment and severity of illness. Clear evidence that increased activity after diagnosis has a positive impact on quality of life supports the notion that weight management and physical activity should be encouraged in the obese cancer patient.

## Limitations

The study included only articles in PubMed and Google scholar, as such, we may miss articles from other search engines. Secondly, we excluded non-English journals which may skew are discussion points and overlooked countries where some of the disease condition mentioned above may be predominant. We are also limited by not including where articles were cited.

## Conclusion

Obesity is a major health care problem in the USA. Given the incidence of obesity in the general population, it is likely that emergency physicians will be involved in the emergency care of obese patients. It is important for all healthcare providers to have an understanding of the medical complications related to obesity in order to maintain appropriate differentials, provide optimal care, and participate in the continued education of our patients.

## Data Availability

Not applicable.

## Abbreviations

ACE: Angiotensin-converting enzyme 2
AD: Alzheimer’s disease
ARB: Angiotensin II receptor blocker
BMI: Body mass index
CDC: Centers for Disease Control and Prevention
DASH: Dietary Approaches to Stop Hypertension
FFA: Free fatty acids
HDL: Hight-density lipoproteins
HDL-C: High-density lipoproteins-C
IHTG: Intrahepatic triglycerides
IHTG: Intrahepatic triglycerides
LDL: Low-density lipoprotein
LPL: Lipoprotein lipase
NAFLD: Non-alcoholic fatty liver disease
NASH: Non-alcoholic steatohepatitis
PCOS: Polycystic ovary syndrome
TG: Triglycerides
VLDL: Very low-density lipoproteins
WHO: World Health Organization
